# Supplementary material for: Post COVID-19 condition after Wildtype, Delta, and Omicron SARS-CoV-2 infection and prior vaccination: Pooled analysis of two population-based cohorts
Source: PLoS One. 2023 Feb 22;18(2):e0281429. doi: 10.1371/journal.pone.0281429 (PMC9946205; doi:10.1371/journal.pone.0281429)
Supplement: S1 Fig — (DOCX) [file pone.0281429.s001.docx]

**S3 Fig. Flowchart of the enrolment, data collection, and inclusion of participants from the Zurich SARS-CoV-2 Cohort and from Phase 5 of the Corona Immunitas seroprevalence study.**
