# Supplementary material for: Post COVID-19 condition after Wildtype, Delta, and Omicron SARS-CoV-2 infection and prior vaccination: Pooled analysis of two population-based cohorts
Source: PLoS One. 2023 Feb 22;18(2):e0281429. doi: 10.1371/journal.pone.0281429 (PMC9946205; doi:10.1371/journal.pone.0281429)
Supplement: S2 Fig — (DOCX) [file pone.0281429.s002.docx]

**S6 Fig. Specific symptoms related to post COVID-19 condition across individuals infected with Wildtype, Delta, and Omicron SARS-CoV-2.** Points represent point estimate and error bars represent 95% Wilson confidence intervals for estimated proportions. GI = gastrointestinal, Prob. = problems, diff. = difficulties.
