# Supplementary material for: Post COVID-19 condition after Wildtype, Delta, and Omicron SARS-CoV-2 infection and prior vaccination: Pooled analysis of two population-based cohorts
Source: PLoS One. 2023 Feb 22;18(2):e0281429. doi: 10.1371/journal.pone.0281429 (PMC9946205; doi:10.1371/journal.pone.0281429)
Supplement: S3 Fig — (DOCX) [file pone.0281429.s003.docx]

**S7 Fig. Results from sensitivity analysis of the association of Delta and Omicron SARS-CoV-2 infection, prior vaccination, and prior infection with post COVID-19 syndrome six months after SARS-CoV-2 infection, and of the association of vaccination with post COVID-19 syndrome stratified by number of received vaccine doses and timing of vaccination.** Panel **A** demonstrates independent associations of Delta and Omicron SARS-CoV-2 infection, prior vaccination, and prior infection with post COVID-19 syndrome six months after SARS-CoV-2 infection, based on a multivariable logistic regression model adjusted for age, sex, presence of comorbidities, initial hospitalization due to COVID-19, and prior infection. Panel **B** and **C** show associations of having received one or two vaccine doses or three doses and of having been vaccinated less than six months prior or six or more months prior to infection, based on multivariable logistic regression model adjusted for age, sex, presence of comorbidities, initial hospitalization due to COVID-19, prior infection, and SARS-CoV-2 variant. CI = confidence interval, OR = odds ratio, Ref. = reference group.
