# Supplementary material for: Post COVID-19 condition after Wildtype, Delta, and Omicron SARS-CoV-2 infection and prior vaccination: Pooled analysis of two population-based cohorts
Source: PLoS One. 2023 Feb 22;18(2):e0281429. doi: 10.1371/journal.pone.0281429 (PMC9946205; doi:10.1371/journal.pone.0281429)
Supplement: S4 Fig — (DOCX) [file pone.0281429.s004.docx]

**S8 Fig. Results from sensitivity analyses regarding the prevalence of post COVID-19 condition six months after infection at different levels of severity.** Panel **A** presents results using symptom count restricted to six symptoms previously found to be in excess among those with post COVID-19 condition compared to the general population (based on Ballouz et al., 2022, https://doi.org/10.1101/2022.06.22.22276746; the six symptoms with the highest excess risk were used for this analysis: fatigue, post-exertional malaise, dyspnoea or shortness of breath, taste or smell alterations, concentration difficulties, and memory problems) as severity categories. Panel **B** shows results using current health status based on EQ-VAS scores as severity categories. Panel **C** demonstrates results using current health status based on EQ-VAS scores as severity categories and restricting the analysis to individuals with no reported comorbidities at baseline to account for potential confounding by impaired baseline health status. Points represent point estimate and error bars represent 95% Wilson confidence intervals for estimated proportions.
