# Supplementary material for: Post COVID-19 condition after Wildtype, Delta, and Omicron SARS-CoV-2 infection and prior vaccination: Pooled analysis of two population-based cohorts
Source: PLoS One. 2023 Feb 22;18(2):e0281429. doi: 10.1371/journal.pone.0281429 (PMC9946205; doi:10.1371/journal.pone.0281429)
Supplement: S5 Fig — (DOCX) [file pone.0281429.s005.docx]

**S12 Fig. Prevalence of specific post COVID-19 condition-related symptoms six months after SARS-CoV-2 infection across symptom clusters, based on a sensitivity analysis assuming five clusters.** Five clusters of individuals with post COVID-19 condition at six months after infection were identified based on multiple correspondence and hierarchical cluster analyses, consisting of individuals with (1) diverse systemic symptoms and lower symptom count, and with (2) predominantly gastrointestinal symptoms or hair loss, (3) neurocognitive, (4) cardiorespiratory, or (5) musculoskeletal symptoms. Panel **A** depicts distributions of specific post COVID-19 condition-related symptoms across clusters. Panel **B** shows the proportion of individuals belonging to each cluster across infections with Wildtype, Delta, and Omicron SARS-CoV-2. Points represent point estimate and error bars represent 95% Wilson confidence intervals for estimated proportions.
