# Supplementary material for: Post COVID-19 condition after Wildtype, Delta, and Omicron SARS-CoV-2 infection and prior vaccination: Pooled analysis of two population-based cohorts
Source: PLoS One. 2023 Feb 22;18(2):e0281429. doi: 10.1371/journal.pone.0281429 (PMC9946205; doi:10.1371/journal.pone.0281429)
Supplement: S1 Table — (DOCX) [file pone.0281429.s007.docx]

**S1 Table. Eligibility criteria, recruitment timeframes, and assessments of the Zurich SARS-CoV-2 Cohort and Phase 5 of the Corona Immunitas seroprevalence study.**

|  | **Zurich SARS-CoV-2 Cohort  (prospectively recruited participants)^a^** | **Corona Immunitas  Zurich & Ticino  Phase 5** |
| --- | --- | --- |
| **Enrolment timeframe** | Aug 6, 2020 – Jan 26, 2021 | Mar 1, 2022 – Mar 31, 2022 |
| **Sample** | Prospective age-stratified (18–64 years, 65+ years) random sample of all individuals with a diagnosed SARS-CoV-2 infection in the canton of Zurich, Switzerland, based on mandatory laboratory reporting to the cantonal public health authorities (Department of Health of the canton of Zurich), contacted via phone and email. | Representative age-stratified (16–29 years, 30–44 years, 45–64 years, 65+ years) random population sample of the population of the cantons of Zurich and Ticino obtained from the Swiss Federal Statistical Office. |
| **Eligibility criteria** | - Laboratory-confirmed SARS-CoV-2 infection between Aug 6, 2020 and Jan 19, 2021 - Residing in the canton of Zurich, Switzerland - Aged 18 years or more - Sufficient knowledge of the German language - Able to follow study procedures - Providing electronic informed consent | - Part of random population sample, responding to study invitation - Resident of the cantons of Zurich or Ticino - Aged 16 years or more - No acute infection with SARS-CoV-2 - Providing written informed consent |
| **Sample size** | N=1106  The sample size was determined based on the objective of evaluating longer-term health status, symptoms, and medical complications or sequelae of SARS-CoV-2 infection across the full severity spectrum of COVID-19. Based on the knowledge available at the time of designing the study, a sample size of 1200 prospectively recruited individuals was deemed sufficient to evaluate differences between different groups of interest (e.g., age, disease severity, and sociodemographic differences) and identify rare sequelae after infection. | N=1894  The sample size was determined based on the objectives of the Corona Immunitas seroprevalence study, aiming to evaluate seroprevalence in Switzerland across social and cultural contexts. Based on the sensitivity and specificity of the used antibody test (98% and 99%, respectively) and an expected seroprevalence of ≥90%, a sample size of 200 individuals per age stratum and canton was determined as sufficient to identify meaningful differences. |
| **Assessments** | **Baseline**   - sociodemographic characteristics - medical comorbidities - initial SARS-CoV-2 infection   **Follow-Up (6 months)^b^**   - current post COVID-19 condition-related symptoms - current health status - medical complications - current health status - new infections - new vaccinations | **Baseline**   - sociodemographic characteristics - medical comorbidities - received vaccinations - past SARS-CoV-2 infections - current post COVID-19 condition-related symptoms - current health status   **Follow-Up (2 and 4 months)**   - current post COVID-19 condition-related symptoms - current health status - new infections - new vaccinations |

**Legend**: **^a^** The Zurich SARS-CoV-2 Cohort consists of two samples of infected individuals. Prospectively recruited participants were enrolled prospectively based on newly reported cases with diagnosed SARS-CoV-2 infection from Aug 6, 2020 to Jan 19, 2021. The study also includes a retrospectively recruited sample for which all individuals diagnosed with SARS-CoV-2 infection between the start of the pandemic in Switzerland (Feb 27, 2020) and start of recruitment (Aug 5, 2020) were contacted. Since testing during the first pandemic wave was restricted in Switzerland and considerations about comparability of populations are warranted, we did not include this sample for the current study. In addition, the cohort includes a case-ascertained sample of close contacts of prospectively recruited individuals, which is also not subject of this study. **^b^** The Zurich SARS-CoV-2 Cohort includes follow-up measurements at 2 weeks, 1 month, 3 months, 6 months, 12 months, and every 6 months up to 36 months. In line with the outcome definition of post COVID-19 condition using a 6 month timeframe, we used data from the 6 month follow-up only to ensure comparability with data from the Corona Immunitas seroprevalence study for the purpose of this study.
