# Supplementary material for: Post COVID-19 condition after Wildtype, Delta, and Omicron SARS-CoV-2 infection and prior vaccination: Pooled analysis of two population-based cohorts
Source: PLoS One. 2023 Feb 22;18(2):e0281429. doi: 10.1371/journal.pone.0281429 (PMC9946205; doi:10.1371/journal.pone.0281429)
Supplement: S2 Table — (DOCX) [file pone.0281429.s008.docx]

**S2 Table. List of the 23 post COVID-19 condition-related symptoms elicited in the Zurich SARS-CoV-2 Cohort and the Corona Immunitas seroprevalence study questionnaires.**

| **Symptoms  (translated)** | **Symptoms  (original wording in German)** |
| --- | --- |
| In the last 7 days, did you have one or several of the following symptoms, which are not related to a chronic condition or an allergy? (Please select all symptoms that you experienced)   - Severe tiredness or exhaustion (fatigue) - Problems breathing or shortness of breath (dyspnoea) - Reduced physical performance (post-exertional malaise) - Cough - Palpitations - Chest pain - Headache - Muscle or bone pain (myalgia) - Joint pain (arthralgia) - Fever - Diarrhea or stool irregularities (gastrointestinal disturbances) - Taste or smell disturbances - Globus sensation or problems swallowing - Tingling or numbness - Trembling - Problems hearing - Vertigo or balance problems - Problems seeing (e.g., flickering, flashes) - Rash - Hair loss - Memory problems - Problems concentrating - Problems sleeping | Hatten Sie in den letzten 7 Tagen eines oder mehrere der folgenden Symptome, welche in keinem Zusammenhang mit einer chronischen Erkrankung oder Allergie stehen? (Bitte geben Sie alle Symptome an, die Sie hatten)   - Starke Müdigkeit oder Erschöpfung (Fatigue) - Atembeschwerden oder Kurzatmigkeit - Reduzierte körperliche Leistungsfähigkeit - Husten - Herzklopfen oder Herzstolpern - Brustschmerzen - Kopfschmerzen - Muskel- oder Gliederschmerzen - Gelenkschmerzen - Fieber - Durchfall oder Stuhlunregelmässigkeiten - Störungen des Geruch- oder Geschmacksinns - Einen Kloss im Hals oder Schwierigkeiten zu schlucken - Kribbeln oder Taubheitsgefühl - Zittern - Hörprobleme - Schwindel oder Balancestörungen - Sehstörungen (z.B. Flimmern, Blitze) - Ausschlag - Haarausfall - Gedächtnisprobleme - Konzentrationsprobleme - Schlafprobleme |
| For each of the symptoms present:  Do you think that this symptom is related to the coronavirus disease "COVID-19" or a complication of the disease? | Für jedes vorhandene Symptom:  Denken Sie, dass dieses Symptom mit der Coronavirus-Krankheit "COVID-19" zusammenhängt oder eine Komplikation dieser Krankheit ist? |
