# Supplementary material for: Post COVID-19 condition after Wildtype, Delta, and Omicron SARS-CoV-2 infection and prior vaccination: Pooled analysis of two population-based cohorts
Source: PLoS One. 2023 Feb 22;18(2):e0281429. doi: 10.1371/journal.pone.0281429 (PMC9946205; doi:10.1371/journal.pone.0281429)
Supplement: S3 Table — (DOCX) [file pone.0281429.s009.docx]

**S4 Table. Detailed participant characteristics of Wildtype, Delta, or Omicron SARS-CoV-2-infected individuals from the Zurich SARS-CoV-2 Cohort and from Phase 5 of the Corona Immunitas seroprevalence study, stratified by study site.**

|  | **Zurich SARS-CoV-2 Cohort** | **Corona Immunitas** | | **Overall** |
| --- | --- | --- | --- | --- |
|  |  | **Ticino** | **Zurich** |  |
|  | **(N=1045)** | **(N=148)** | **(N=157)** | **(N=1350)** |
| **Timeframe of diagnosis** | Aug 5, 2020 – Jan 19, 2021 | Jul 15, 2021 – Feb 24, 2022 | Jul 26, 2021 – Feb 25, 2022 | Aug 5, 2020 – Feb 25, 2022 |
| **Median follow-up (IQR; days)** | 183.5 (182–186) | 187 (165–196) | 179 (162–193) | 183 (182–186) |
| **Age, median (IQR)** | 51 (35–66) | 41 (30–54) | 43 (31–54) | 48 (34–63) |
| **Age group** |  |  |  |  |
| 16-29 | 158 (15.1%) | 37 (25.0%) | 35 (22.3%) | 230 (17.0%) |
| 30-44 | 243 (23.3%) | 50 (33.8%) | 49 (31.2%) | 342 (25.3%) |
| 45-64 | 346 (33.1%) | 49 (33.1%) | 54 (34.4%) | 449 (33.3%) |
| 65+ | 298 (28.5%) | 12 (8.1%) | 19 (12.1%) | 329 (24.4%) |
| **Female sex** | 530 (50.7%) | 96 (64.9%) | 83 (52.9%) | 709 (52.5%) |
| **Presence of chronic comorbidity** | 308 (29.5%) | 23 (15.5%) | 20 (12.7%) | 351 (26.0%) |
| **Smoking status** |  |  |  |  |
| Non-smoker | 625 (60.1%) | 98 (66.2%) | 103 (65.6%) | 826 (61.4%) |
| Ex-smoker | 282 (27.1%) | 31 (20.9%) | 31 (19.7%) | 344 (25.6%) |
| Smoker | 133 (12.8%) | 19 (12.8%) | 23 (14.6%) | 175 (13.0%) |
| *Missing* | *5* | *0* | *0* | *5* |
| **BMI, median (IQR; kg/m2)** | 24.2 (21.9–26.6) | 23.2 (21.3–25.4) | 23.5 (21.6–26.3) | 24.0 (21.7–26.5) |
| **Highest education** |  |  |  |  |
| None or mandatory school | 41 (3.9%) | 11 (7.5%) | 7 (4.5%) | 59 (4.4%) |
| Vocational training or specialised baccalaureate | 438 (42.2%) | 84 (57.1%) | 70 (44.9%) | 592 (44.1%) |
| Higher technical school or college | 276 (26.6%) | 9 (6.1%) | 27 (17.3%) | 312 (23.2%) |
| University | 284 (27.3%) | 43 (29.3%) | 52 (33.3%) | 379 (28.2%) |
| *Missing* | *6* | *1* | *1* | *8* |
| **Employment status** |  |  |  |  |
| Employed | 668 (64.2%) | 28 (18.9%) | 29 (18.6%) | 725 (53.9%) |
| Retired | 256 (24.6%) | 79 (53.4%) | 102 (65.4%) | 437 (32.5%) |
| Student | 50 (4.8%) | 13 (8.8%) | 19 (12.2%) | 82 (6.1%) |
| Unemployed or other | 67 (6.4%) | 28 (18.9%) | 6 (3.8%) | 101 (7.5%) |
| *Missing* | *4* | *0* | *1* | *5* |
| **Monthly household income** |  |  |  |  |
| <6'000 CHF | 330 (33.2%) | 51 (37.2%) | 47 (30.9%) | 428 (33.4%) |
| 6'000 - 12'000 CHF | 443 (44.6%) | 61 (44.5%) | 59 (38.8%) | 563 (43.9%) |
| >12'000 CHF | 221 (22.2%) | 25 (18.2%) | 46 (30.3%) | 292 (22.8%) |
| *Missing* | *51* | *11* | *5* | *67* |
| **Hospitalised due to COVID-19** | 44 (4.2%) | 0 (0.0%) | 2 (1.3%) | 46 (3.4%) |
| **SARS-CoV-2 variant** |  |  |  |  |
| Wildtype | 1045 (100%) | 0 (0.0%) | 0 (0.0%) | 1045 (77.4%) |
| Delta | 0 (0.0%) | 42 (28.4%) | 57 (36.3%) | 99 (7.3%) |
| Omicron | 0 (0.0%) | 106 (71.6%) | 100 (63.7%) | 206 (15.3%) |
| **Prior vaccination** | 0 (0.0%) | 112 (76.7%) | 120 (77.4%) | 232 (17.2%) |
| **Vaccine doses^a^** |  |  |  |  |
| 1-2 doses | – | 87 (77.7%) | 86 (71.7%) | 173 (74.6%) |
| 3 doses | – | 25 (22.3%) | 34 (28.3%) | 59 (25.4%) |
| **Time since last vaccine dose^a^** |  |  |  |  |
| <6 months | – | 87 (77.7%) | 93 (77.5%) | 180 (77.6%) |
| ≥6 months | – | 25 (22.3%) | 27 (22.5%) | 52 (22.4%) |
| **Type of vaccines received^a^** |  |  |  |  |
| mRNA | – | 112 (100%) | 119 (99.2%) | 231 (99.6%) |
| Adenovirus vector | – | 0 | 1 (0.8%) | 1 (0.4%) |
| **Prior SARS-CoV-2 infection** | 0 (0.0%) | 16 (10.8%) | 20 (12.7%) | 36 (2.7%) |

**Legend:** BMI = body mass index, CHF = Swiss Francs, IQR = interquartile range. **^a^** Percentages among those that have received at least one vaccine dose prior to infection.
