# Supplementary material for: Post COVID-19 condition after Wildtype, Delta, and Omicron SARS-CoV-2 infection and prior vaccination: Pooled analysis of two population-based cohorts
Source: PLoS One. 2023 Feb 22;18(2):e0281429. doi: 10.1371/journal.pone.0281429 (PMC9946205; doi:10.1371/journal.pone.0281429)
Supplement: S4 Table — (DOCX) [file pone.0281429.s010.docx]

**S5 Table. Comparison of participant characteristics of Phase 5 Corona Immunitas seroprevalence study participants in Zurich and Ticino, Switzerland, with non-included individuals (stratified by infection status).**

|  | **Included participants** | **Non-included individuals** | | |
| --- | --- | --- | --- | --- |
|  | **(N=305)** | **Ever infected (N=634)** | **Never infected (N=956)** | **All (N=1590)** |
| **Age, median (IQR)** | 43 (30–54) | 43 (30–59) | 55 (37–69) | 51 (34–66) |
| **Sex** |  |  |  |  |
| female | 179 (58.7%) | 364 (57.4%) | 508 (53.2%) | 872 (54.9%) |
| male | 126 (41.3%) | 269 (42.4%) | 445 (46.6%) | 714 (44.9%) |
| other/diverse | 0 (0.0%) | 1 (0.2%) | 2 (0.2%) | 3 (0.2%) |
| *Missing* | *0* | *0* | *1* | *1* |
| **Presence of chronic comorbidity** | 43 (14.1%) | 140 (22.1%) | 288 (30.2%) | 428 (27.0%) |
| *Missing* | *0* | *0* | *3* | *3* |
| **Smoking status** |  |  |  |  |
| Non-smoker | 201 (65.9%) | 404 (63.7%) | 563 (59.2%) | 967 (61.0%) |
| Ex-smoker | 62 (20.3%) | 135 (21.3%) | 230 (24.2%) | 365 (23.0%) |
| Smoker | 42 (13.8%) | 95 (15.0%) | 158 (16.6%) | 253 (16.0%) |
| *Missing* | *0* | *0* | *5* | *5* |
| **BMI, median (IQR; kg/m2)** | 23.3 (21.5–26.0) | 23.9 (21.5–26.6) | 24.1 (21.7–27.4) | 24.0 (21.5–27.1) |
| *Missing* | *0* | *1* | *3* | *4* |
| **Highest education** |  |  |  |  |
| None or mandatory school | 18 (5.9%) | 48 (7.6%) | 94 (10.0%) | 142 (9.0%) |
| Vocational training or specialised baccalaureate | 154 (50.8%) | 291 (46.1%) | 458 (48.5%) | 749 (47.6%) |
| Higher technical school or college | 36 (11.9%) | 118 (18.7%) | 157 (16.6%) | 275 (17.5%) |
| University | 95 (31.4%) | 174 (27.6%) | 235 (24.9%) | 409 (26.0%) |
| *Missing* | *2* | *3* | *12* | *15* |
| **Employment status** |  |  |  |  |
| Employed | 57 (18.8%) | 161 (25.5%) | 402 (42.5%) | 563 (35.7%) |
| Retired | 181 (59.5%) | 344 (54.5%) | 411 (43.4%) | 755 (47.9%) |
| Student | 32 (10.5%) | 70 (11.1%) | 71 (7.5%) | 141 (8.9%) |
| Unemployed | 34 (11.2%) | 56 (8.9%) | 62 (6.6%) | 118 (7.5%) |
| *Missing* | *1* | *3* | *10* | *13* |
| **Monthly household income** |  |  |  |  |
| <6'000 CHF | 98 (33.9%) | 227 (38.2%) | 399 (44.8%) | 626 (42.2%) |
| 6'000 - 12'000 CHF | 120 (41.5%) | 233 (39.2%) | 345 (38.8%) | 578 (38.9%) |
| >12'000 CHF | 71 (24.6%) | 135 (22.7%) | 146 (16.4%) | 281 (18.9%) |
| *Missing* | *16* | *39* | *66* | *105* |
| **Ever vaccinated** | 233 (76.4%) | 571 (90.1%) | 896 (93.7%) | 1467 (92.3%) |

**Legend:** BMI = body mass index, CHF = Swiss Francs, IQR = interquartile range.
