# Supplementary material for: Post COVID-19 condition after Wildtype, Delta, and Omicron SARS-CoV-2 infection and prior vaccination: Pooled analysis of two population-based cohorts
Source: PLoS One. 2023 Feb 22;18(2):e0281429. doi: 10.1371/journal.pone.0281429 (PMC9946205; doi:10.1371/journal.pone.0281429)
Supplement: S5 Table — (DOCX) [file pone.0281429.s011.docx]

**S9 Table. Results from sensitivity analyses of the association of SARS-CoV-2 variant and vaccination with severity of post COVID-19 condition based on multinomial logistic regression models, using symptom count restricted to six symptoms previously found to be in excess among those with post COVID-19 condition compared to the general population as severity categories.** Excess symptoms were defined based on Ballouz et al., 2022 (https://doi.org/10.1101/2022.06.22.22276746), using the six symptoms with the highest excess risk for this analysis (fatigue, post-exertional malaise, dyspnoea or shortness of breath, taste or smell alterations, concentration difficulties, and memory problems).

| **Characteristic** | **0-1 symptoms** | | **2 symptoms** | | **3+ symptoms** | |
| --- | --- | --- | --- | --- | --- | --- |
|  | **OR (95% CI)** | **p-value** | **OR (95% CI)** | **p-value** | **OR (95% CI)** | **p-value** |
| **Non-vaccinated Wildtype** | Ref. |  | Ref. |  | Ref. |  |
| **Non-vaccinated Delta** | 0.51 (0.15–1.77) | 0.29 | 0.56 (0.06–4.97) | 0.60 | 2.35 (0.69–7.97) | 0.17 |
| **Non-vaccinated Omicron** | 1.03 (0.37–2.89) | 0.96 | n.e. | n.e. | 1.41 (0.27–7.43) | 0.68 |
| **Vaccinated Delta** | 0.48 (0.19–1.22) | 0.12 | 0.34 (0.05–2.56) | 0.30 | 1.03 (0.30–3.50) | 0.96 |
| **Vaccinated Omicron** | 0.37 (0.19–0.71) | 0.0027 | 0.38 (0.11–1.35) | 0.14 | 0.65 (0.24–1.74) | 0.39 |

**Legend:** CI = confidence interval, n.e. = not estimable, OR = odds ratio, Ref. = reference group.
