# Supplementary material for: Post COVID-19 condition after Wildtype, Delta, and Omicron SARS-CoV-2 infection and prior vaccination: Pooled analysis of two population-based cohorts
Source: PLoS One. 2023 Feb 22;18(2):e0281429. doi: 10.1371/journal.pone.0281429 (PMC9946205; doi:10.1371/journal.pone.0281429)
Supplement: S6 Table — (DOCX) [file pone.0281429.s012.docx]

**S10 Table. Results from sensitivity analyses of the association of SARS-CoV-2 variant and vaccination with severity of post COVID-19 condition based on multinomial logistic regression models, using current health status based on EQ-VAS scores as severity categories.**

| **Characteristic** | **Mild  (EQ-VAS >70)** | | **Moderate  (EQ-VAS 51-70)** | | **Severe  (EQ-VAS ≤50)** | |
| --- | --- | --- | --- | --- | --- | --- |
|  | **OR (95% CI)** | **p-value** | **OR (95% CI)** | **p-value** | **OR (95% CI)** | **p-value** |
| **Non-vaccinated Wildtype** | Ref. |  | Ref. |  | Ref. |  |
| **Non-vaccinated Delta** | 0.81 (0.32–2.10) | 0.67 | n.e. | n.e. | 2.46 (0.30–19.9) | 0.40 |
| **Non-vaccinated Omicron** | 0.50 (0.14–1.73) | 0.27 | 2.02 (0.40–10.3) | 0.40 | 3.52 (0.43–28.9) | 0.24 |
| **Vaccinated Delta** | 0.47 (0.20–1.10) | 0.08 | 1.44 (0.41–5.00) | 0.57 | n.e. | n.e. |
| **Vaccinated Omicron** | 0.41 (0.23–0.72) | 0.002 | 0.49 (0.13–1.84) | 0.29 | 0.41 (0.05–3.18) | 0.39 |

**Legend:** CI = confidence interval, n.e. = not estimable, OR = odds ratio, Ref. = reference group, VAS = visual analogue scale.
