# Supplementary material for: Post COVID-19 condition after Wildtype, Delta, and Omicron SARS-CoV-2 infection and prior vaccination: Pooled analysis of two population-based cohorts
Source: PLoS One. 2023 Feb 22;18(2):e0281429. doi: 10.1371/journal.pone.0281429 (PMC9946205; doi:10.1371/journal.pone.0281429)
Supplement: S7 Table — (DOCX) [file pone.0281429.s013.docx]

**S11 Table. Results from sensitivity analyses of the association of SARS-CoV-2 variant and vaccination with severity of post COVID-19 condition based on multinomial logistic regression, using current health status based on EQ-VAS scores as severity categories and restricting the analysis to individuals with no reported comorbidities at baseline to account for potential confounding by impaired baseline health status.**

| **Characteristic** | **Mild  (EQ-VAS >70)** | | **Moderate  (EQ-VAS 51-70)** | | **Severe  (EQ-VAS ≤50)** | |
| --- | --- | --- | --- | --- | --- | --- |
|  | **OR (95% CI)** | **p-value** | **OR (95% CI)** | **p-value** | **OR (95% CI)** | **p-value** |
| **Non-vaccinated Wildtype** | Ref. |  | Ref. |  | Ref. |  |
| **Non-vaccinated Delta** | 0.68 (0.22–2.05) | 0.49 | n.e. | n.e. | n.e. | n.e. |
| **Non-vaccinated Omicron** | 0.36 (0.08–1.61) | 0.18 | 2.73 (0.53–14.0) | 0.23 | 4.47 (0.52–38.3) | 0.17 |
| **Vaccinated Delta** | 0.55 (0.21–1.42) | 0.22 | 1.98 (0.42–9.37) | 0.39 | n.e. | n.e. |
| **Vaccinated Omicron** | 0.45 (0.25–0.83) | 0.010 | 0.78 (0.20–3.09) | 0.72 | 0.64 (0.08–5.33) | 0.68 |

**Legend:** CI = confidence interval, n.e. = not estimable, OR = odds ratio, Ref. = reference group, VAS = visual analogue scale.
