# Supplementary material for: Post COVID-19 condition after Wildtype, Delta, and Omicron SARS-CoV-2 infection and prior vaccination: Pooled analysis of two population-based cohorts
Source: PLoS One. 2023 Feb 22;18(2):e0281429. doi: 10.1371/journal.pone.0281429 (PMC9946205; doi:10.1371/journal.pone.0281429)
Supplement: S8 Table — (DOCX) [file pone.0281429.s014.docx]

**S14 Table. Participant characteristics among infected individuals with post COVID-19 condition in the four clusters identified through cluster analysis.** Identified clusters were composed of (1) diverse systemic, (2) cardiorespiratory, (3) neurocognitive, and (4) musculoskeletal symptoms.

|  | **Cluster 1** | **Cluster 2** | **Cluster 3** | **Cluster 4** |
| --- | --- | --- | --- | --- |
|  | **(N=219)** | **(N=47)** | **(N=23)** | **(N=19)** |
| **Age, median (IQR)** | 52 (37–66) | 49 (34.5–60) | 56 (42–67.5) | 59 (47.5–76.5) |
| **Female sex** | 119 (54.3%) | 33 (70.2%) | 18 (78.3%) | 11 (57.9%) |
| **Presence of chronic comorbidity** | 75 (34.2%) | 15 (31.9%) | 12 (52.2%) | 10 (52.6%) |
| **Smoking status** |  |  |  |  |
| Non-smoker | 120 (55.3%) | 27 (57.4%) | 14 (60.9%) | 8 (42.1%) |
| Ex-smoker | 65 (30.0%) | 7 (14.9%) | 6 (26.1%) | 6 (31.6%) |
| Smoker | 32 (14.7%) | 13 (27.7%) | 3 (13.0%) | 5 (26.3%) |
| *Missing* | *2* | *0* | *0* | *0* |
| **Variant** |  |  |  |  |
| Wildtype | 192 (87.7%) | 39 (83.0%) | 18 (78.3%) | 15 (78.9%) |
| Delta | 9 (4.1%) | 4 (8.5%) | 4 (17.4%) | 0 (0.0%) |
| Omicron | 18 (8.2%) | 4 (8.5%) | 1 (4.3%) | 4 (21.1%) |
| **Prior vaccination** | 17 (7.8%) | 7 (14.9%) | 1 (4.3%) | 3 (15.8%) |
| **Symptom count, median (IQR)** | 1 (1–2) | 4 (3–6) | 5 (4–7) | 5 (4–9) |
| 1-2 symptoms | 179 (81.7%) | 10 (21.3%) | 2 (8.7%) | 0 (0.0%) |
| 3-5 symptoms | 39 (17.8%) | 22 (46.8%) | 11 (47.8%) | 11 (57.9%) |
| 6+ symptoms | 1 (0.5%) | 15 (31.9%) | 10 (43.5%) | 8 (42.1%) |

**Legend:** IQR = interquartile range.
